# Case Report: Alpha6 Integrin Disorder Presenting in Childhood with Nail Dysplasia and Onycholysis But No History of Fragile or Bullous Skin Changes

**DOI:** 10.1055/s-0044-1791804

**Published:** 2024-10-16

**Authors:** Alayna N. Zalesny, Sarah Gunter, Charles A. Williams

**Affiliations:** 1 OU Health Pediatric Specialties Clinic, Oklahoma Children's Hospital, Oklahoma City, Oklahoma, United States; 2 Division of Genetics and Metabolism, Department of Pediatrics, University of Florida College of Medicine, Gainesville, Florida, United States

**Keywords:** ITGA6, onycholysis, nail dysplasia

## Abstract

We report a 7-year-old girl born with pyloric atresia but without congenital epidermolysis bullosa or skin fragility. Nail dysplasia developed at age 8 months and throughout childhood she suffered from onycholysis and mild nail hypertrophy. Whole-exome sequencing demonstrated biallelic mutations in alpha6 integrin (ITGA6): p. Q139* and R153W. ITGA6 normally forms a protein heterodimer with beta4 integrin (ITGB4), and this dimer participates in anchoring the basal skin cells to the extracellular matrix. Biallelic mutations in each gene are well known to cause epidermolysis bullosa and pyloric atresia. However, this child had ostensibly normal skin without any evidence of skin fragility. In a literature search, we identified 11 cases involving ITGA6 mutations, and all had epidermolysis skin changes. Thus, this case adds to the reported phenotype of ITGA6 disease since it is the first to show absence of an epidermolysis bullosa phenotype in the setting of pyloric atresia and nail dysplasia.

## Introduction

Integrins are a large family of proteins predominantly acting in the extracellular matrix as structural and signaling proteins. Two of these integrins, beta 4 (ITGB4) and alpha6 (ITGA6), form a heterodimer that promotes the structural integrity of skin. This heterodimer is part of a larger protein complex, the hemidesmosome, that anchors the basal skin cells to the extracellular matrix. 1

Biallelic mutations in ITGB4 are well-known to cause congenital pyloric atresia and a neonatal, junctional type of epidermolysis bullosa (JT-EB). 2 3 4 In JT-EB, the basement membrane shows disruption at the lamina lucida region, an area of the extracellular matrix where integrin heterodimers protrude. More recently, ITGA6 clinical cases have been reported to also cause the same intestinal and skin phenotype. 4 5 6 7 8 9 10 11 12 13 The epidermolysis bullosa in these cases is frequently lethal in neonates and infants, due to severe mucocutaneous fragility and aplasia cutis congenita. 3

The case presented here, although having congenital pyloric atresia, did not show any manifestations of epidermolysis bullosa. Accordingly, the clinicians discounted the possibility that either of these integrins were the cause of her problems. Whole-exome sequencing, however, identified biallelic mutations in ITGA6.

## Case Report

This is a 7-year-old girl referred to the genetics clinic for evaluation of fingernail and toe dysplasia. She was seen at age 5 years by a dermatologist and no diagnosis was made although a variant of Clouston type of ectodermal dysplasia was considered.

She was born vaginally at 36 weeks' gestation after a normal prenatal period. Birth weight was 2,038 g, head circumference 33.5 cm, and length 46.5 cm. Gastric obstruction was diagnosed on day 1 of life and she subsequently had surgical repair of complete pyloric atresia. During the neonatal period there were no problems with any type of skin fragility or bullous skin formations. Her nails were not noted to be abnormal at birth but at 8 months of age some of her nails began to fall off. The mother said that, for no apparent reason, they would “turn black” then fall off and there was an apparent normal nail underneath. This problem has continued to date on her fingers and toes. In early childhood she began to have thickening to her thumbs and great toenails, but the other nails were not thickened.

Family history is essentially negative; there are two normally developed full siblings. The parents are normal; no one in the kindred has problems with either nail development, sparse hair, or abnormal skin or thickening of the skin of palms and soles. She had no history of urinary tract infections. Intellectual development and motor milestones were normal.

**Fig. 1:**
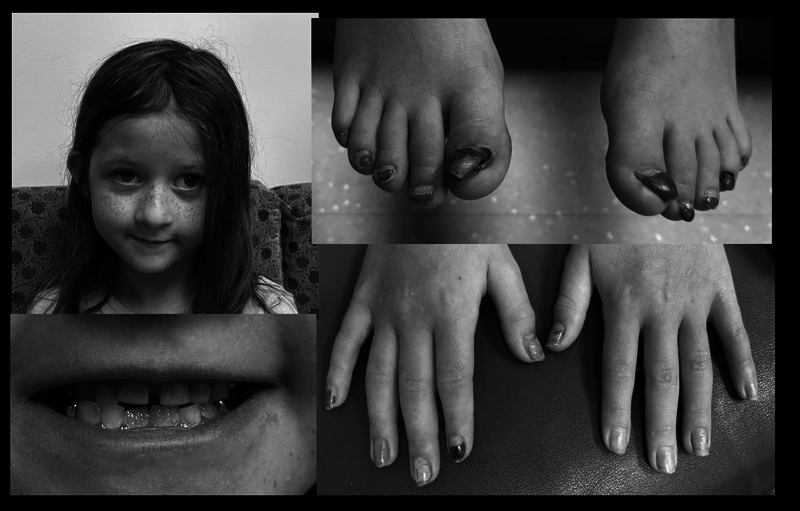
Normal facial picture of the girl with normally textured hair. The toes, especially the great toenails, demonstrate pronounced arthrogryposis. Fingernails are less involved with only mild hypertrophy of the thumbnails. At the time of this photograph, no onycholysis was present. Dental view shows apparent normally erupting lower mandibular incisors, normal upper incisors with apparent normal dental enamel involvement. Carries are partly evident by the capped tooth.

On examination, she was a well-developed, happy girl with prominent freckles over the malar region of her face. Her height and weight were at the 30th percentile and she appeared normocephalic. Her hair was normally textured and not sparse. The oral exam disclosed relatively normal upper incisors and lateral incisors. She has two newly erupting central mandibular incisors. Posteriorly, she has multiple caps over many of the molars: these are primary teeth. Her teeth generally have good coloration and there is nothing suggesting an enamel defect. Extremity exam shows fingers and nails as described above and shown in Fig. 1 . The most abnormal are the great toenails which are hypertrophic and have a horn-like shape; one of them is almost ready to fall off and has an underlying discolored toenail. Skin exam is normal without any scarring or bruising or abnormal texture. She appeared to have normal sweating. Chest and abdomen are normal except she has a well-healed transverse surgical scar from her previous pyloric atresia repair.

## Genetic Testing

Whole-exome sequencing was performed by GeneDx (United States) from buccal tissue specimens of the child and mother, using an Illumina (United States) platform. Two mutations in ITGA6 were identified: p. Gln139Ter (c.415 C > T), in exon 4 and p. Arg153Trp (c.457 C > T), also in exon 4. The former mutation is a frameshift causing protein termination, interpreted as being likely pathogenic, and the latter mutation is a missense, interpreted as being a variant of uncertain significance (VUS). The mother carried only the Gln139Ter mutation. The father was not available for testing.

## Discussion

The nail dystrophy in this patient seems best described as a combination of generalized chronic onycholysis with thickening of the thumbnails and toenails (e.g., nail arthrogryposis). The history of all or part of the nail spontaneously turning black before falling off presumably indicates some degree of nontraumatic subungual hemorrhage in the nail bed, beneath the nail plate. This region is formed by a thin layer of epithelium, only 2 or 3 cell layers thick, residing between the nail bed and the underlying connective tissue. 14 While nail dystrophy can be associated with JT-EB, our review of all previously reported ITGA6 cases ( Table 1 ) did not find any report of either subungual hemorrhage or onycholysis. There are nine congenital genetic nail dysplasia syndromes identified in the Online Mendelian Inheritance in Man (OMIM) database (refer to OMIM #161050 15 for discussion of genetic heterogeneity of these disorders). While onycholysis is occasionally noted, it is usually not the major manifestation in any of these nail dysplasia conditions. Information of nail problems is difficult to determine from the 11 case reports that we identified (see Table 1 ) because of the young age of affected infants. The oldest surviving infant was 3 months old and of the 11 cases, 4 died by intrauterine death or were aborted. Of the 11 cases there is only a case where it was noted that no nail abnormalities were present.

**Table 1:** Clinical features in all previously reported ITBA6 cases

Case report patients	Age	Skin features	Nails dysplasia	Pyloric atresia	Mutation	Other
Allegra et al, 2003	At birth (died at 26 days)	Aplasia cutis, blistering with friction	Not reported on	Present	*ITGA6* , c.286C > T (p.S47L), homozygous	Microtia, consanguineous parents
Gache et al, 1998; also, Lépinard et al, 2000	Abortion	Aplasia cutis with complete detachment of the integument	Not reported on	Present	*ITGA6* homozygous, c.1764C > T; (also reported as c.1618C > T) p.R540X	Abnormal limbs with fisted hands, overlapping fingers, malposition of first toe, hypoplastic ears
Ghazzawi and Fatma, 2023 (one family)	At birth (died at 2 days)	Bullous eruptions	Not reported on	Duodenal atresia	Presumed c.1688dup	Consanguineous parents
	IUFD at 32 weeks	Blistering of skin	Not reported on	Not reported on	Presumed c.1688dup	Depressed nasal bridge, low-set ears, consanguineous parents
	IUFD at 30 weeks	Blistering of skin	Not reported on	Present	*ITGA6* , c.1688dup (p.Met563Ilefs*10), homozygous	Polyhydramnios, dilated right renal pelvis and ureter, low-set ears Consanguineous parents
Masunaga et al, 2017	At birth	Blisters and skin erosion	Not reported on	Present	*ITGA6* , c.3876 > T and c.2506-1G > C	Patent ductus arteriosus
Pulkkinen et al, 1997 (one family)	At birth (died at 40 days)	Blistering of skin	Not reported on	Duodenal atresia	Presumed c.1856 + 1G > T	
	Abortion	Blistering of skin	Not reported on	Duodenal atresia	Presumed c.1856 + 1G > T	
	At birth (died at 29 days)	Blistering of skin	Not reported on	Not reported on	*ITGA6* , c.1856 + 1G > T, homozygous	Bilateral cleft lip and palate
Ruzzi et al, 1997	At birth (died at 23 days)	Blisters and skin erosion, cutaneous aplasia	Not reported on	Present	*ITGA6* , c.791delC, homozygous	Small and malformed pinnae of both ears, esophageal stenosis
Schumann et al, 2013	At birth (died at age 3 months)	Generalized blistering, widespread erosions	No nail anomalies	Present	*ITGA6* , c.388-5T > G, homozygous	Dilated renal pelvis, thickened bladder wall, hematuria, cholestasis
This case	7 years	No history of skin fragility or bullous changes	Onycholysis, nail hyperplasia gryposis	Present	p. Gln139Ter (c.415C > T) and p. Arg153Trp (c.457C > T)	Multiple dental caries

Abbreviation: IUFD, intrauterine fetal death.

This child had multiple carries significant enough to require inpatient dental surgery to place numerous caps over most of the molars. The teeth themselves did not appear to have overt enamel defects, so it is unclear what the origin of her multiple carries are. Schuman et al reported that 4 of 7 patients with IGTB4 disease also had enamel defects, tooth loss, or multiple carries. 4 The family history in our case does not disclose any other members who have such severe carries as is reported here in this child.

We believe that the missense VUS is likely to be pathogenic in our case even though it has not been reported yet in ITGA6 databases. It has also not been observed at significant frequency in general population databases. The mother carries only the frameshift mutation, indicating that the VUS is in a trans-allelic configuration, so the mechanism for the clinical disorder is expected to be autosomal recessive due to compound heterozygous mutations. A different ITGA6 missense VUS, also at amino acid position 153, has been reported in the clinical variation (ClinVar) database (United States; https://www.ncbi.nlm.nih.gov/clinvar/ ): p. Arg153Gln (c.458G > A). In that ClinVar case, the substituted glutamine was a more conservative amino acid change than the tryptophan substitution in our case. Since the whole-exome sequencing is otherwise negative, and the family history does not suggest any other skin disorder, the likelihood of coincidentally finding biallelic ITGA6 mutations in a child with congenital pyloric atresia and nail dystrophy, seems extremely low. For these reasons, we conclude that the ITGA6 missense VUS in our case is operative in some way to account for the observed phenotype.

Since in our case we were not able to test to determine if any ITGA6 protein is expressed and/or persists without decay, we can only speculate as to what might be the mechanism for the phenotype in this child. The frameshift mutation is presumed to create at least functional ITGA6 haploinsufficiency. If the missense mutation leads to a completely transcribed protein without ribonucleic acid decay, then formation of the normal heterodimer between ITGA6 and ITGB4 could in some way still be flawed and create a dominant negative effect. The missense mutation does lie in the beta-propeller region of the ITGA6 heavy chain which is a probable ligand binding and signaling domain of the protein. 1 5 It is also possible that a transcribed missense-mutated protein could not have any effect on heterodimer formation but instead lead to abnormal interaction with an adjacent protein in the extracellular matrix. A candidate for this might be BPAG2 (also known as collagen 17A1) which is known to bind, in the extracellular matrix, to ITG6A. 16 Biallelic mutations in BPAG2 also cause a JT-EB 17 associated with nail dystrophy, but we found no reports of pyloric atresia in the BPAG2 disorder.

## Conclusion

The nail dystrophy observed in this girl expands the clinical spectrum of skin and connective tissue manifestations associated with biallelic mutations in ITGA6. As more cases are reported for this rare autosomal recessive condition, it will become clearer as to what extent there are milder dermatological manifestations of the autosomal recessive ITGA6 disorder.
